# Correlation between driving-related skill and alcohol use in young-adults from six European countries: the TEN-D by Night Project

**DOI:** 10.1186/1471-2458-11-526

**Published:** 2011-07-01

**Authors:** Roberta Siliquini, Fabrizio Bert, Francisco Alonso, Paola Berchialla, Alessandra Colombo, Axel Druart, Marcin Kedzia, Valeria Siliquini, Daniel Vankov, Anita Villerusa, Lamberto Manzoli, TEN-D Group (TEN-D by Night Group)

**Affiliations:** 1 Department of Public Health, University of Turin, Italy; 2 INTRAS, Universitat de València-UVEG, Spain; 3 Responsible Young Drivers, Brussels, Belgium; 4 Prezes Fundacji "Kierowca Bezpieczny", Warsaw, Poland; 5 S&T soc. coop., Turin, Italy; 6 Open Youth, Sofia, Bulgaria; 7 Dep. of Public Health and Epidemiology, Riga Stradins University, Latvia; 8 Section of Epidemiology and Public Health, University "G. d'Annunzio" of Chieti, Italy

## Abstract

**Background:**

Only few studies with small experimental samples investigated the impact of psychoactive substances on driving performance. We conducted a multicenter international cross-sectional study to evaluate the correlation between alcohol use and driving-related skill as measured by brake reaction time (RT).

**Methods:**

Before and after the entrance into randomly selected recreational sites from six European countries, all subjects aged 16-35 years, owning a driver license, were asked to compile a structured socio-demographic questionnaire and measure RT (SimuNomad3 driving simulator), breath alcohol concentration (BAC; Drager Alcoltest), and drug use (Oratect III saliva test, only at the exit). Mixed regression modeling was used to evaluate the independent association between RT and alcohol concentration or drug use.

**Results:**

Before the entrance into the recreational site, 4534 subjects completed all assessments and composed the final sample. Their mean age was 23.1 ± 4.2y; 68.3% were males; 54.7% had BAC > 0 g/L (assumed alcoholics); 7.5% declared illegal drug assumption (mostly cannabis). After the exit, 3019 also completed the second assessment: 71.7% showed BAC > 0 g/L. Controlling for age, gender, educational level, occupation, driver license years, and drug use, BAC was positively associated with RT, achieving significance, however, only when BAC was higher than 0.49 g/L. Significant interaction terms were found between BAC and female gender or drug use, with highest RTs (> 1 sec.) recorded among drug users with BAC > = 1 g/L.

**Conclusions:**

This field study confirms previous experimental data on the negative impact of alcohol use on driving-related skill, supporting regulations and educational campaigns aimed at discouraging driving after consumption of psychoactive substances.

## Background

Between 1995 and 2004, according to Traffic Safety Facts almost 73,500 young people (aged 16-24) died in road accidents in 14 European countries [1,2], and road crashes are considered the first cause of death in people aged 10-24 years in developed countries [3].

Previous researches have indicated that younger age, inexperience in driving and consumption of alcohol and illegal drugs, especially in the week-end evenings, are the main risk factors of traffic crashes [4-10].

As regards alcohol and illegal drug consumption, several studies focused on their correlation with simulated driving performance [11-17]. Driving performance was influenced by a number of factors, such as tracking, vigilance, divided attention tasks, visual functions and driving skills. Several studies reported the reaction time as a proxy of driving-related skill [6,18,19]. Such studies, however, were mostly based on small samples, in controlled experimental or laboratory settings [14-17]. As an example, Marczinski et al. investigated driving performance in a sample of 40 college students [11], while Liguori has conducted a study on 18 adults [19]. Other studies included only people admitted to hospital emergency departments following a crash [20-26].

To date, however, no studies with large samples evaluated the correlation between consumption of psychoactive substances and driving performance in natural settings, and although the negative impact of alcohol and illegal drugs on cognitive ability has long been recognized, their relative contribution on driving performance remains unclear [18,27-30].

The TEN-D (Dark, Dance, Disco, Dose, Drugs, Drive, Danger, Damage, Disability, Death) by Night is an international multicentric cross-sectional survey, conducted on a large sample of young people in six European countries [31], aimed at investigating the relationship between driving-related skill, as measured by brake reaction time, and alcohol and illegal drugs assumption.

## Methods

### Study design and population

The study protocol has been described in detail elsewhere [31]. In brief, the TEN-D by Night project is a multidisciplinary, multicentric, international, cross-sectional survey. The project was endorsed by the European Commission Public Health Executive Agency and it was carried out in six European countries: Italy, Belgium/Netherlands, Bulgaria, Poland, Spain.

The TEN-D survey collected data on subjects aged 16 to 35 years, who owned a driving license and attended recreational sites during weekend nights. The recreational meeting places were selected on the basis of official regional lists and according to the willingness of the club's owners. At the entry of the recreational site, each participant was administered a first questionnaire by survey staff, who was previously trained and was composed by at least six operators in each intervention. The first questionnaire collected information on demographic and socioeconomic status, medication consumption, alcohol and illegal drug consumption, and driving habits. At the exit from each recreational site, a second questionnaire was administered to each participant, investigating alcohol and illegal drug consumption during the event, intention to drive, and opinion about the intervention. The entrance (first) and exit (second) questionnaires were developed based on validated questionnaires [32,33], which were translated into the national language of each of the participating country.

Also, at the entry of the recreational site, we evaluated the breath alcohol concentration (BAC -proxi of alcohol consumption) and the reaction time (RT -proxi of driving-related skill) of each participant, using:

- the Drager Alcoltest 6510 (the measurement results are given in g/L and, according to the manufacturer, their standard deviation is ± 1.7% of the measurement value) [34];

- the driving simulator SimuNomad3 Ecrans, provided with the Software SCAM 03, to measure the latency time to release the gas pedal, along with the latency time to press the brake pedal, after the appearance of a fence on a screen. Reaction time was defined as the latency to apply the brake after appearance of a barrier in a driving simulator) [35].

At the exit of the recreational site, the same tests were performed again. In addition, each participant was subjected to a saliva test: the Oratect III Oral Fluid Drug Screen Device; a one-step chromatographic immunoassay device employed for the qualitative simultaneous detection of multiple illegal drugs: cocaine, metamphetamine/MDMA, THC, amphetamine, opiates, benzodiazepines [36].

An informed consent was obtained by all participants in each intervention and the TEN-D by Night project was approved by the ethics committee of the coordinating centre (the Central Ethics Committee of the Catholic University of the Sacred Heart in Rome, Italy).

### Data analysis

The sample size was calculated using the Schlesselmann formula for known population sizes [37], and computation details have been reported previously [31].

Analyses were primarily aimed at identifying potential predictors of reaction time, with particular emphasis on the possible role of BAC and drug use. Importantly, however, drug assumption was used only as a confounding factor, for several reasons: it was only self-reported at the entry; although it was tested at the exit, the adopted test had very high specificity (98%) but low sensitivity (53%) [38]; finally the test cutoffs for some drugs were higher than those requested by current guidelines [39]. Therefore, drug use data could only be used as a covariate into multivariate models, and the estimates on its prevalence and association with reaction time, although explored, could be unreliable and were not considered as primary aims of the study.

Firstly, mean (SD) reaction times were reported stratifying for several variables including age-class (16-19; 20-24; 25-29 and 30-35 years), gender, BAC in g/l (arbitrarily categorized in five classes: 0.0; 0.01-0.19; 0.20-0.49; 0.50-0.99; > = 1.00), educational level, occupation, and driving license years (for either automobiles or motorcycles). Because drug use may confound the association with the above variables and reaction time, the univariate results were additionally stratified according to drug use, and both data from the first and second assessment (before and after the stay in the recreational site) were showed. Additionally, we also tested the potential association between reaction time and variables related to past drug or alcohol use, and driving history (i.e. previous car accident due to driving after drinking).

Secondly, the independent association between reaction time (dependent variable) and blood alcohol concentration was evaluated using several multivariate approaches. First, separate random-effects regression models (using country as the cluster variable) were fitted for the observations collected before the entry and after the exit from the recreational site. Given that, as expected, the results of these models were substantially concordant, we fitted a single model using all observations. In other terms, the n_1 _BAC values recorded at the entry were used together with the n_2 _BAC values recorded at the exit, obtaining n_2_+n_2 _observations. Clearly, the same process was applied to reaction times at entry and exit, and drug use (at the entry, the data from the questionnaire were used, while the data from the drug test were used at the exit). We then used a mixed regression model, with two cluster levels: country and subject identification code (to account for repeated observations) [40]. We again found results that were similar to those from previous models, and the likelihood ratio test confirmed the need for a multilevel approach as opposed to normal regression. We thus reported only the estimates from the mixed model to avoid redundancy. All recorded covariates were included into the model a priori, except for those variables related to past use of alcohol or drug and driving history, which were included only if significant at the 0.10 level. BAC, age and educational level were included as continuous variables rather than ordinal ones based upon the Wald test results. Interaction and higher power terms were tested for all covariates, and only those achieving significance were retained. Outlier analysis for the separate regression models was based upon studentized residuals and DFITS and Cook's D influence measure. We found 201-242 influential observations (up to 5.3%) according to the model and methodology (i.e. n = 228 using DFITS > 0.85 in the "entry" model), and repeated all analyses excluding them. We did not observe substantial changes in any covariates, and thus used the whole dataset. We assumed an independent correlation structure for both cluster levels in the mixed model, however we repeated the model indicating an exchangeable correlation structure, with marginal increases in standard errors and no qualitative change. Expectedly, some degree of multicollinearity was observed between the number of alcohol units declared by each subject and their BAC (as assessed by the Drager Alcoltest) either before or after the stay inside the local (Spearman rho: 0.61 and 0.67, respectively). The number of units declared was less relevant for the analysis and excluded from multivariate models. Notably, either BAC at the entry and at the exit remained highly significant in all models even when the number of alcohol units declared was included. Finally, with the exception of occupation (n = 138), drug use testing at the exit of the recreational site (n = 273), and previous detain because of driving (n = 141) missing items were less than 15 for all variables and no missing imputation technique was thus adopted.

As a confirmatory secondary analysis, we carried out a different model to investigate the independent association between the change in reaction time (dependent variable) and the change in BAC between the entry and exit from the recreational site. The analysis was based upon a random-effect regression model (country as the only cluster variable) and was clearly limited to those accepting the alcohol test also at the exit from the recreational site. Criteria for model building were the same as above: all variables included into the multivariate analysis on reaction time were forced to entry into the model.

A two-tailed p-value of 0.05 was considered significant for all analyses, which were carried out using Stata, version 10.1 (Stata Corp., College Station, TX, USA, 2007).

## Results

### Characteristics of the sample and alcohol and drug use

Overall, 4534 subjects compiled the first questionnaire, performed the first Alcoltest and had their reaction time measured through the Driving simulator. Their mean age was 23.1y (SD = 4.2), and 68.3% were males (Table 1).

**Table 1:** Characteristics of the sample

	Before (n = 4534)	After (n = 3019)	No response (n = 1515)	
	%	%	%	P*
Male gender	68.3	70.3	64.4	< 0.001
				
*Age class, y*				
16-19	23.1	24.5	20.5	
20-24	46.2	45.0	48.8	
25-29	20.8	20.0	22.2	
30-35	9.9	10.5	8.5	0.001
				
*Educational level*				
None	3.0	2.8	3.2	
Mandatory school	14.6	13.9	16.1	
High school	52.8	57,2	44.1	
Diploma	29.6	26.1	36.5	< 0.001
				
*Occupation*				
Student	47.2	47.1	47.3	
Employed	45.1	45.1	45.0	
Unemployed	7.7	7.8	7.7	0.9
				
*Driving licence years*				
No licence	10.8	13.2	6.1	
< = 2	26.7	26.7	26.6	
3-5	25.0	24.1	26.8	
> 5	37.5	36.0	40.5	< 0.001
				
Declaring alcohol assumption	58.3	77.0	69.5 φ	0.015 §
				
Number of alcohol units declared **				
0	44.6	23.0	34.2 φ	--
1-2	26.3	36.3	23.3 φ	--
> = 3	29.1	40.7	42.5 φ	0.011 §
				
*Type of drinks declared*				
None	41.7	23.0	30.5 φ	--
Beer (only beer)	27.6 (16.1)	--	28.2 (9.7) φ	--
Wine (only)	12.2 (4.9)	--	18.1 (5.7) φ	--
Super alcoholics (only)	21.4 (9.8)	--	24.7 (6.8) φ	--
Cocktails (only)	7.8 (2.8)	--	11.5 (2.9) φ	--
Pops (only)	15.0 (6.6)	--	32.9 (13.8) φ	--
Mixing alcoholics	18.1	--	30.7 φ	< 0.001 §
				
*Alcohol test: BAC, g/l*				
0.0	45.3	28.3	35.8 φ	--
0.01-0.19	12.5	10.4	13.2 φ	--
0.20-0.49	16.3	18.1	17.1 φ	--
0.50-0.99	17.1	26.3	21.5 φ	--
> = 1.00	8.8	16.9	12.4 φ	0.009 §
				
Declaring (any) drug assumption	7.5	4.5	13.7 φ	< 0.001 §
				
*Drug type declared*				
Cannabis	6.3	3.3	11.6 φ	0.004 §
Cocaine	1.0	0.8	2.2 φ	--
Amphetamines	0.4	0.8	0.5 φ	--
Benzodiazepine	0.2	0.1	0.1 φ	--
Opiates	0.1	0.0	0.3 φ	--
Other	0.1	0.2	0.2 φ	--
				
*Drug test: any drug (missing = 273)*	*--*	*6.3*	*--*	*--*
Cannabis	--	4.3	--	--
Cocaine	--	1.4	--	--
Amphetamines	--	1.1	--	--
Benzodiazepine	--	0.4	--	--
Opiates	--	0.4	--	--
				
Mean age in years (SD)	23.1 (4.2)	23.1 (4.3)	23.0 (4.1)	0.9 §

BAC = Blood Alcohol concentration. * Chi-squared test to compare the sample who answered and did not answer after the stay inside the local. ** One alcohol unit = 125 ml of wine or 330 ml of beer or 40 ml of spirit. φ Referred to the data before the entrance in the local only. § p-values are referred to the comparison between those refusing the second evaluation (n = 1515) and those accepting it (n = 3019), and are based upon the parameters of the first assessment.

Before the entry in the recreational site, more than half of the participants declared they assumed alcohol, and 29.1% affirmed they drank 3 or more alcohol units (18.0% mixed different types of alcoholics). In agreement with declarations, the BAC was 0.0 g/l only in 45.3% of the subjects; while 25.9% and 8.8% of the subjects showed a BAC higher than 0.50 and 1 g/l, respectively. A minority reported illegal drug use (7.5%); mostly cannabis (6.3%).

At the exit from the recreational site, only 3019 of the 4534 participants accepted to perform the second assessment (66.6%). The main characteristics of those who accepted and refused the second evaluation are separately reported in Table 1. The majority of responders declared they assumed alcohol inside the recreation site (77.0%), and 40.7% drank 3 or more alcohol units. The percentages of those with BAC higher than 0.50 and 1.00 g/l were 43.2% and 16.9%, respectively. Only 4.5% of the subjects declared illegal drug use inside the recreational site. Clearly, the interpretation of all exit data should be cautious because of the above mentioned relevant proportion of subjects who refused the second assessment. Indeed, and unsurprisingly, those who refused the second evaluation, as compared to those who accepted, were more likely to declare alcohol and drug assumption at the first evaluation, to have mixed alcoholics, and to show a BAC > 1.00 g/l (all p < 0.02). Additional information on the characteristics of the subjects with BAC equal or higher than 0.50 g/l, both at the first and the second assessment, have been provided in the additional file 1.

When the focus moved to past drug or alcohol use, the prevalence of users largely increased: 91.2% and 31.1% declared they assumed alcohol and cannabis, respectively, in the last month. Driving misbehaviors in the recent past were not infrequent: 19.7% declared they had driven while inebriate in the last month; 8.2% had their driving license suspended; 4.6% caused a car accident because of drinking and 2.4% were arrested because of driving after drinking in the last year. More detailed results have been reported in the additional file 1.

### Reaction Time

The mean reaction time of the sample, overall and stratified by several variables, is reported in Table 2. Because entry and exit data were similar, they can be discussed together. In the majority of the sample who did not use illegal drugs (n = 4195 before the entrance; n = 2789 after), the reaction time was slightly higher in females, with low educational level, unemployed. As regards the relationship between BAC and reaction time, the latter progressively increased with increasing BAC after zero g/l, and the highest reaction time was invariably noted among those with BAC equal or more than 1 g/l. Unexpectedly, however, those with BAC = 0 g/l showed a higher mean reaction time as compared to those with BAC < 1 g/l. Notably, the above pattern was observed in both males and females.

**Table 2:** Reaction time according to blood alcohol concentration (BAC) and other characteristics of the sample

	**Before**, no drugs	**Before**, drug users	**After**, no drugs*	**After**, drug users**
	(n = 4193)	(n = 337)	(n = 2542)	(n = 210)
*Overall*	0.72 (0.32)	0.81 (0.62)	0.72 (0.31)	0.78 (0.32)
Males	0.69 (0.31)	0.79 (0.67)	0.71 (0.32)	0.79 (0.34)
Females	0.77 (0.34)	0.89 (0.46)	0.74 (0.29)	0.76 (0.23)
				
*BAC, g/l (All)*				
0.0	0.73 (0.29)	0.68 (0.27)	0.75 (0.28)	0.73 (0.36)
0.01-0.19	0.65 (0.27)	0.80 (0.71)	0.67 (0.24)	0.72 (0.19)
0.20-0.49	0.68 (0.28)	0.81 (0.53)	0.67 (0.24)	0.74 (0.25)
0.50-0.99	0.72 (0.34)	0.74 (0.65)	0.70 (0.37)	0.76 (0.27)
> = 1.00	0.84 (0.50)	1.13 (0.84)	0.76 (0.35)	0.88 (0.37)
				
*BAC, g/l (Males)*				
0.0	0.70 (0.29)	0.67 (0.28)	0.75 (0.28)	0.75 (0.42)
0.01-0.19	0.63 (0.28)	0.78 (0.82)	0.65 (0.24)	0.73 (0.23)
0.20-0.49	0.65 (0.26)	0.78 (0.58)	0.65 (0.24)	0.73 (0.25)
0.50-0.99	0.70 (0.36)	0.73 (0.72)	0.69 (0.39)	0.73 (0.28)
> = 1.00	0.80 (0.40)	1.09 (0.88)	0.75 (0.35)	0.91 (0.39)
				
*BAC, g/l (Females)*				
0.0	0.77 (0.30)	0.73 (0.27)	0.75 (0.29)	0.66 (0.19)
0.01-0.19	0.70 (0.25)	0.86 (0.14)	0.71 (0.25)	0.69 (0.12)
0.20-0.49	0.76 (0.29)	0.87 (0.43)	0.70 (0.25)	0.78 (0.25)
0.50-0.99	0.76 (0.25)	0.80 (0.26)	0.76 (0.30)	0.84 (0.24)
> = 1.00	0.97 (0.69)	1.23 (0.74)	0.79 (0.35)	0.73 (0.25)
				
*Age class, y*				
16-19	0.71 (0.34)	1.09 (1.10)	0.69 (0.31)	0.79 (0.30)
20-24	0.72 (0.32)	0.71 (0.32)	0.73 (0.33)	0.74 (0.32)
25-29	0.73 (0.32)	0.79 (0.49)	0.72 (0.28)	0.87 (0.36)
30-35	0.71 (0.27)	0.72 (0.23)	0.71 (0.29)	0.78 (0.28)
				
*Educational level*				
None	1.01 (0.46)	0.95 (0.43)	1.03 (0.37)	1.03 (0.39)
Mandatory school	0.78 (0.31)	0.94 (0.78)	0.77 (0.30)	0.94 (0.40)
High school	0.68 (0.31)	0.74 (0.37)	0.68 (0.30)	0.73 (0.28)
Diploma	0.73 (0.31)	0.81 (0.79)	0.73 (0.31)	0.70 (0.22)
				
*Occupation*				
Student	0.70 (0.34)	0.85 (0.70)	0.69 (0.31)	0.78 (0.33)
Employed	0.73 (0.30)	0.78 (0.53)	0.74 (0.31)	0.77 (0.29)
Unemployed	0.80 (0.33)	0.84 (0.69)	0.81 (0.34)	0.84 (0.39)
				
*Driving licence years*				
No licence	0.65 (0.34)	0.78 (0.75)	0.64 (0.27)	0.70 (0.26)
< = 2	0.75 (0.33)	1.02 (0.95)	0.73 (0.29)	0.85 (0.35)
3-5	0.74 (0.35)	0.74 (0.33)	0.76 (0.35)	0.80 (0.32)
> 5	0.70 (0.29)	0.72 (0.37)	0.71 (0.31)	0.74 (0.30)

All data are reported as means (SD). M = Males; F = Females. * Excluding (or including) those with drug test missing. ** Including both those declaring drug use inside the local and those found positive to the Oratect III test, independently on their declaration.

By contrast, the above U-shaped trend in the relationship between BAC and reaction time was not observed among those declaring drug use: reaction time progressively increased with increasing BAC even when subjects with BAC = 0 g/l were considered. Importantly, and expectedly, the highest mean reaction times were observed among those who used drugs and showed BAC values equal or above 1 g/l (1.09 seconds among males; 1.23 seconds among females).

### Multivariate analysis

The results of the mixed regression models investigating potential independent predictors of reaction time have been reported in Table 3. Even after controlling for age, gender, educational level, occupation, driving license years, drug use and detain due to driving after drinking, reaction time significantly increased with increasing BAC. Importantly, because of the unexpected U-shaped relationship between BAC and reaction time at univariate analysis, the possibility of higher power terms (non significant in any model), transformation and categorization for BAC was extensively explored. When analyses were adjusted for other covariates, (and in particular driving license years), the relationship between BAC and reaction time became approximately linear, and BAC might be included in the model as a continuous variable. Indeed, univariate analyses were confounded by the higher proportion of subjects who obtained the driving license from 2 to 5 years and who were less likely to drink (and be accordingly classified into the category BAC = 0 g/l -data not shown).

**Table 3:** Results of the multivariate analysis (mixed regression model) evaluating potential predictors of reaction time

	First model			Second model *		
	Regression coefficient	(95% CI)	p	Regression coefficient	(95% CI)	p
BAC, 0.1 g/l increase	0.015	(0.012; 0.018)	< 0.001	--		
						
BAC classes						
0.0 (ref. category)	--			0	--	--
0.01-0.19	--			-0.016	(-0.037; 0.005)	0.144
0.20-0.49	--			0.018	(-0.002; 0.037)	0.068
0.50-0.99	--			0.050	(0.031; 0.070)	< 0.001
> = 1.00	--			0.196	(0.147; 0.244)	< 0.001
						
Drug use	-0.007	(-0.041; 0.026)	0.7	-0.015	(-0.054; 0.024)	0.5
BAC*drug use	0.015	(0.006; 0.024)	0.002	0.010	(0.005; 0.014)	< 0.001
						
Male gender	-0.065	(-0.042; -0.088)	< 0.001	-0.076	(-0.054; -0.097)	< 0.001
BAC*Male gender	-0.011	(-0.004; -0.019)	0.003	-0.008	(-0.004; -0.011)	< 0.001
						
Age, 1-year increase	0.003	(-0.001; 0.006)	0.10	0.003	(-0.001; 0.006)	0.090
						
Educational level, ordinal	0.001	(-0.012; 0.014)	0.9	0.001	(-0.013; 0.014)	0.9
						
Occupation						
Student (ref. category)	0	--	--	0	--	--
Employed	0.013	(-0.010; 0.035)	0.3	0.011	(-0.011; 0.034)	0.3
Unemployed	0.075	(0.040; 0.110)	< 0.001	0.074	(0.039; 0.109)	< 0.001
						
Driving licence years						
No licence (ref. category)	0	--	--	0	--	--
< = 2	0.090	(0.057; 0.124)	< 0.001	0.092	(0.059; 0.126)	< 0.001
3-5	0.068	(0.033; 0.103)	< 0.001	0.069	(0.034; 0.104)	< 0.001
> 5	0.043	(0.005; 0.082)	0.027	0.046	(0.007; 0.083)	0.019
						
Arrested because of driving after drinking in the last year	0.161	(0.101; 0.220)	< 0.001	0.158	(0.098; 0.217)	< 0.001

* The second mixed model includes the same covariates of the first; the only difference is the inclusion of dummy variables for BAC classes (categorization).

In the same table, we are also showing the results of a second, very similar model, in which BAC was treated categorically and separate coefficients are reported for each BAC class (given that the potential existence of a BAC cutoff after which reaction time does increase significantly was also an hypothesis of extreme interest). It can also be noted that the differences in reaction time between the subjects with BAC = 0 g/l and those with BAC < 0.5 g/l were no more significant at multivariate analysis. Although the relationship between BAC and reaction time is approximately linear, only after 0.5 g/l of BAC the increase in reaction time achieved significance.

Interestingly, when adjusting for BAC and other covariates, illegal drug use was not significantly associated with an increase in reaction time. However, the interaction term between BAC and drug use was strongly significant, indicating that the negative effect of alcohol increases when also drugs are assumed.

Also the interaction between gender and BAC was highly significant, suggesting that alcohol influence on reaction time was smaller in males than females, who also showed a higher reaction time per se. Finally, the other independent predictors of a rise in reaction time were unemployment and owning a driving license. Interestingly, there seemed to be a inverse trend between reaction time and the number of years of driving experience, but the direct comparison among driving license categories was not or only borderline significant (data not shown).

Table 4 shows the results of the secondary multivariate analysis, investigating potential predictors of the change in reaction time between the entry and exit from the recreational site. The results of the first model were substantially confirmed: an increase in BAC was significantly associated with an increase in reaction time; and the increase of BAC between the entry and the exit from the recreational site caused a lower augment of reaction time among the males, who also showed a lower reaction time than females (although of borderline significance). This analysis was based upon 3019 subjects only (those accepting to participate to both first and second evaluations) and was adjusted for the same covariates that were also included in the above mixed model.

**Table 4:** Results of the multivariate analysis (random-effect regression) evaluating potential predictors of the change in reaction time before and after the stay inside the recreational site (dependent variable: time change = time after -time before)

	Regression coefficient	(95% CI)	p
Change in BAC before and after the stay inside the recreational site (BAC after -BAC before), 0.1 g/l increase	0.019	(0.013; 0.025)	< 0.001
			
Male gender	0.025	(-0.001; 0.052)	0.052
BAC change*Male gender	-0.010	(-0.016; -0.003)	0.004
			
Drug use	0.029	(-0.010; 0.069)	0.15
BAC*drug use	0.001	(-0.009; 0.011)	0.9
			
Age, 1-year increase	0.001	(-0.003; 0.005)	0:6
			
Educational level, ordinal	0.009	(-0.006; 0.025)	0.2
			
Occupation			
Student (ref. category)	0	--	--
Employed	-0.009	(-0.036; 0.018)	0.5
Unemployed	-0.019	(-0.060; 0.023)	0.4
			
Driving licence years			
No licence (ref. category)	0	--	--
< = 2	0.006	(-0.032; 0.044)	0.8
3-5	0.015	(-0.025; 0.053)	0.5
> 5	0.019	(-0.023; 0.062)	0.4
			
Arrested because of driving after drinking in the last year	0.001	(-0.001; 0.002)	0.5

## Discussion

Driving performance depends on several factors pertaining to driving expertise and level of attention, of which reaction time is considered a valid indicator [41]. Several studies documented that reaction time can be influenced by alcohol or psychoactive drug use, but uncertainties remain of the quantification of both the associations [18,27-30]. To our knowledge, this is the first study providing a precise estimate of the influence of alcohol intake on reaction time from a large sample of young-adult individuals from different European countries, in natural recreation settings.

Adjusting for age, gender, educational level, occupation, driving license years and illegal drug use, we found a significant and independent worsening in reaction time with increasing alcohol intake (BAC). Clearly, the increase in reaction time was highest when BAC was over 1.00 g/l, especially in females and in those subjects who also used drugs (significant interactions with alcohol intake were observed for both factors). Importantly, when compared to those who did not assume or already metabolized alcohol (BAC equal to zero), reaction time was significantly higher only when BAC value overcome 0.50 g/l, indicating that below such a threshold reaction time does not seem to be substantially altered. Given the recent diffusion of energetic drinks, in which caffeine or other stimulants are mixed with alcohol, and may confound the real strength (and shape) of the association between alcohol and reaction time, we analyzed separately the subjects who assumed drinks that could (cocktails, alcohol pops, or mixed) or could not (beer only, wine only, super-alcoholics only) be mixed with energetic substances, finding however no differences at both univariate and multivariate analyses (data not shown). Although a more precise analysis was not possible due to the lack of further details on the assumed drinks, it is unlikely the observed lack of significance in the association between 0 < BAC < 0.50 g/l and reaction time increase is entirely biased. In any case, this finding should not be misinterpreted as a demonstration that moderate alcohol use does not affect driving, as it has been repeatedly documented that other components affecting driving performance such as danger perception are altered even at moderate blood alcohol concentrations [42-44].

When the interaction term between drug use and alcohol concentration was included in multivariate analysis, the association between drug use per se and reaction time was no longer significant. However, we already mentioned several reasons (no test at the entry, low sensitivity of the exit test) why the present data on drug use should be interpreted with extreme caution. Also, we had no data on drug concentration, thus it may be possible that the observed lack of association was simply due to the assumption of very low quantities of drugs by most of the participants. In any case, as for moderate alcohol levels (BAC < 0.5 g/l), such finding should not be misinterpreted: even if drug assumption was not independently related with a worsening in reaction time, its use significantly increased the negative effect of alcohol.

All of the above findings confirm and expands those from previous studies on the negative impact of alcohol and illegal drugs on cognitive abilities (cognitive faculty is, in effect, the first to be impaired by drinking, resulting in deteriorated performance in tasks related to attention, memory, logical reasoning and visual perception [18,27-30]) and driving-related-skills and performance (drug use among vehicle drivers increases the risk for a road trauma accident requiring hospitalization [7,14,17,28]).

Besides the negative interaction between alcohol concentration and female gender, our analyses also showed a longer reaction time in females than males, confirming previous findings on driving-related skills differences by gender [45,46].

Concerning other predictors of reaction time, unemployed subjects (vs students and employed people), as well as those with a driving license (vs no driving license), showed a longer reaction time. The foster finding is of complex interpretation, while it may be hypothesized that people with no driving license were kept the simulator test more seriously than those already driving, who might have been more relaxed because of the higher confidence in their reaction in such a situation. Similarly complex to interpret is the finding of an independent and strongly significant association between a longer reaction time and having been arrested because of driving after drinking. In fact, it may be supposed that these subjects would have been more careful in performing their examination. Certainly, the present results suggest the need for additional research on these subjects, for whom a reaction time test might even be supposed for license returning.

Other results of interest are the very high proportion of subjects who had already drank before the entry in the recreational site (54.7%; 8.8% with BAC > = 1.00 g/l), and the even higher proportion of those who drank after the stay in the local (71.7%; 16.9% with BAC > = 1.00 g/l). Also, 6.3% of the participants were found to have assumed illegal drugs after the stay despite widespread prohibitions in all participating countries. Moreover, the latter findings are certainly underestimated because of the low sensitivity of the exit test and the large proportion of subjects who refused the second assessment (33.4%). In fact, these subjects showed a higher proportion of alcohol and drug users at the first assessment, and their refusal might have been motivated in many cases by the awareness of having assumed drugs or drunk too much to perform well. In addition, all data on the prevalence of alcohol and drug use must be interpreted with extra caution also because the study was not designed to this aim and the sample cannot be considered representative of the whole young-adult population. As an example, and although several differences in the studied populations, Miller et al. reported a drug use prevalence as high as 26% in patrons of clubs featuring electronic music dance [47].

A last mention deserves false-reporting. Alcohol assumption was both self-reported and objectively evaluated (through the Alcoltest). The same was made for drug use at the exit from the recreational site. Therefore, we had the opportunity to roughly estimate the amount of false declarations in both contexts. More than a fifth (20.6%) of those declaring no alcohol assumption at the entry showed BAC > 0 g/l, while only 2.9% of those declaring no drug use were found positive to the Drug test (data not shown). However, they represented 43.7% of all the subjects that were found positive to the test (which, again, had low sensitivity). Moreover, our participants knew they were going to be objectively assessed, thus the percentage of false reporting is likely to be substantially lower than in surveys where consumption is only self-reported. Clearly, these findings confirm previous researches suggesting that such a limitation should be taken into account when interpreting the results from studies on alcohol or drug consumption that are only based upon self-reporting [48].

This study has some limitations that deserve discussion. First, as above mentioned, the sample cannot be considered representative of neither the whole population of young-adults nor the subset of subjects attending recreational sites, because of the voluntary recruitment of participants and the opportunistic selection of recreational sites. However, if such a bias is likely to influence the results on alcohol and drug use prevalence, there are no reasons to believe that it may affect the association between alcohol concentration or drug use and reaction time.

Second, driving simulator performance may not accurately reflect what actually happens in road driving where other distraction factors may influence cognitive abilities [49,50]. Although the few studies that directly compared the two situations used excursions out of lane rather than reaction time, they reported a high level of agreement between simulator results and real driving-related skill [51,52].

Third, we did not investigate in detail the potential influence of the different types of alcoholics or drugs consumed. However, for such analyses to be meaningful (especially for drug type), a much larger sample size would have been required, and their reliability would still be limited by their dependence on self-reporting. In any case, we found no differences in reaction time according to the vast categories of alcoholics that we used (beer, wine, spirits, cocktails, alcohol-pops, mixed), but these results are of limited utility given that there is huge variability in the alcohol content within the same class of drinks.

Fourth, the design of the study is cross-sectional, therefore we were able to document only associations between variables, not the presence of a causal relationship. However, we also investigated the variation in reaction time according to the variation in alcohol concentration (between the entry and the exit from the recreational site), finding a significant positive association between BAC levels and reaction time, thus confirming the common sense of a causal role of alcohol on reaction time changes, over and above the influence of other factors (some of which were adjusted into the analysis, such as age and gender, while some others were not, such as fatigue [53,54]).

## Conclusion

In conclusion, in this international multicenter survey, based upon objective evaluations in naturalistic settings, we found that reaction time significantly increases with increasing breath alcohol concentration, especially when also illegal drugs are assumed. Importantly, the difference in reaction time between subjects with zero alcohol concentration and those with higher alcohol levels achieved significance only after the cutoff of 0.50 g/l, being highest after 1.00 g/l. Although reaction time is only one of the determinants of driving-related skill, our findings strongly support National legislations and widespread educational campaigns aimed at discouraging driving after drinking or drug assumption.

## Abbreviations

BAC: Breath Alcohol Concentration; RT: Reaction Time; MDMA: 3,4-Methylenedioxymethamphetamine; THC: Tetrahydrocannabinol; SD: Standard Deviation.

## Competing interests

The authors declare that they have no competing interests.

## Authors' contributions

All the authors and TEN-D Group contributed to the conception, design and implementation of TEN-D by Night study. RS, FB and LM wrote the present manuscript. All authors read and approved the final manuscript.

## Pre-publication history

The pre-publication history for this paper can be accessed here:

http://www.biomedcentral.com/1471-2458/11/526/prepub

## Supplementary Material

Additional file 1 **Characteristics of the sample according to blood alcohol concentration**. The table contains the characteristics of the sample according to BAC. Variables considered are gender, age class, educational level, occupation, driving license years, alcohol and drugs consumption declared and drugs tested. Reaction time according to past alcohol and drug use and driving history in the overall sample. The table contains the reaction time values (means - SD) according to past alcohol and drug use and driving history in the overall sample (n = 4534). Click here for file
