# Supplementary material for: Correlation between driving-related skill and alcohol use in young-adults from six European countries: the TEN-D by Night Project
Source: BMC Public Health. 2011 Jul 1;11:526. doi: 10.1186/1471-2458-11-526 (PMC3145590; doi:10.1186/1471-2458-11-526)
Supplement: Additional file 1 — Characteristics of the sample according to blood alcohol concentration. The table contains the characteristics of the sample according to BAC. Variables considered are gender, age class, educational level, occupation, driving license years, alcohol and drugs consumption declared and drugs tested. Reaction time according to past alcohol and drug use and driving history in the overall sample. The table contains the reaction time values (means - SD) according to past alcohol and drug use and driving history in the overall sample (n = 4534). [file 1471-2458-11-526-S1.DOC]

Online supplemental Table 1. Characteristics of the sample according to blood alcohol concentration.

|  | Before  BAC>=0.5g/l  (n=1175) | After  BAC>=0.5g/l  (n=1305) |
| --- | --- | --- |
|  | % | % |
|  |  |  |
| *Gender* |  |  |
| Male | 27.9 | 47.0 |
| Female | 21.7 | 34.1 |
|  |  |  |
| *Age class, y* |  |  |
| 16-19 | 24.2 | 49.7 |
| 20-24 | 28.9 | 43.7 |
| 25-29 | 25.5 | 38.5 |
| 30-35 | 16.6 | 34.9 |
|  |  |  |
| *Educational level* |  |  |
| None | 20.1 | 37.7 |
| Mandatory school | 27.0 | 38.6 |
| High school | 23.6 | 44.4 |
| Diploma | 30.3 | 43.9 |
|  |  |  |
| *Occupation* |  |  |
| Student | 28.9 | 48.3 |
| Employed | 23.6 | 37.1 |
| Unemployed | 25.3 | 44.6 |
|  |  |  |
| *Driving licence years* |  |  |
| No licence | 23.4 | 53.3 |
| <=2 | 24.0 | 41.2 |
| 3-5 | 27.9 | 43.8 |
| >5 | 26.7 | 40.7 |
|  |  |  |
| *Number of alcohol units declared *** |  |  |
| 0 | 10.5 | 6.4 |
| 1-2 | 16.9 | 34.9 |
| >=3 | 57.6 | 71.5 |
|  |  |  |
| *Declaring (any) drug assumption* |  |  |
| No | 24.4 | 41.6 |
| Yes | 45.8 | 60.9 |
|  |  |  |
| *Drug type declared* |  |  |
| Cannabis | 43.9 | 63.6 |
| Cocaine | 66.0 | 70.8 |
| Amphetamines | 60.0 | 45.8 |
| Benzodiazepine | 40.0 | 33.3 |
| Opiates | 50.0 | 0.0 |
| Other | 83.3 | 50.0 |
|  |  |  |
| *Drug test: any drug (missing=273)* | *--* | *61.6* |
| Cannabis | -- | 59.7 |
| Cocaine | -- | 69.2 |
| Amphetamines | -- | 65.5 |
| Benzodiazepine | -- | 30.0 |
| Opiates | -- | 60.0 |
|  |  |  |
| Mean age in years (SD) | 22.7 (3.8) | 22.7 (4.1) |
| Mean age in years BAC<0.5 (SD) | 23.2 (4.4) | 23.5 (4.5) |
|  |  |  |

Online supplemental Table 2. Reaction time according to past alcohol and drug use and driving history in the overall sample (n=4534).

|  | Reaction time |
| --- | --- |
|  | Mean (SD) |
|  |  |
| *Driving after heavy drinking in the last month* |  |
| No | 0.71 (0.33) |
| Yes (n=895) | 0.76 (0.40) |
|  |  |
| *Previous car accident because of drinking* |  |
| No | 0.72 (0.33) |
| Yes (n=198) | 0.77 (0.42) |
|  |  |
| *Penalties for speed limit violation in the past* |  |
| None | 0.72 (0.35) |
| One (n=1033) | 0.73 (0.37) |
| More than one (n=303) | 0.76 (0.33) |
|  |  |
| *Driving licence suspended in the past* |  |
| Never | 0.72 (0.35) |
| Once (n=299) | 0.72 (0.39) |
| More than once (n=56) | 0.71 (0.26) |
|  |  |
| *Arrested because of driving after drinking in the last year* |  |
| No | 0.72 (0.32) |
| Yes (n=103) | 0.97 (0.91) |
|  |  |
| *Alcoholics assumption in the last month* |  |
| Never | 0.73 (0.30) |
| Once-twice (n=1020) | 0.73 (0.32) |
| Once a week (n=1736) | 0.72 (0.33) |
| 3-4 times a week (n=1000) | 0.71 (0.36) |
| Daily (n=397) | 0.77 (0.52) |
|  |  |
| *Cannabis assumption in the last month* |  |
| Never | 0.72 (0.33) |
| Once-twice (n=326) | 0.74 (0.29) |
| Once a week (n=608) | 0.73 (0.36) |
| 3-4 times a week (n=168) | 0.69 (0.45) |
| Daily (n=248) | 0.80 (0.60) |
|  |  |
